# Supplementary material for: Deep Learning with Quantitative Features of Magnetic Resonance Images to Predict Biochemical Recurrence of Radical Prostatectomy: A Multi-Center Study
Source: Cancers (Basel). 2021 Jun 21;13(12):3098. doi: 10.3390/cancers13123098 (PMC8234539; doi:10.3390/cancers13123098)
Supplement: Supplementary file 1 [file cancers-13-03098-s001.zip › cancers-1169990-supplementary.pdf]

cancers

Article

Supplementary Material: Deep Learning with Quantitative Features of Magnetic Resonance Images to Predict Biochemical Recurrence of Radical Prostatectomy: A Multi-Center Study

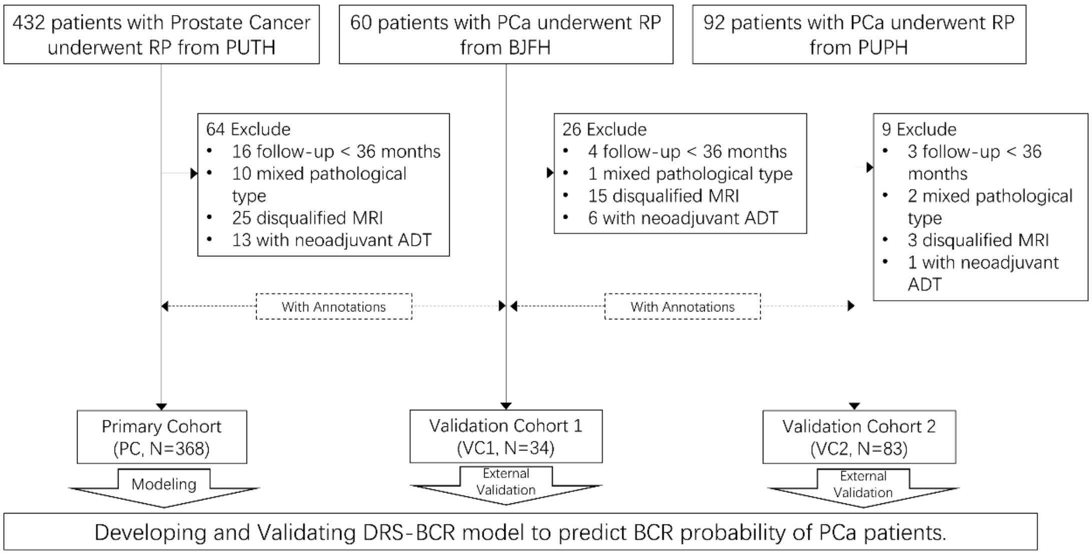

Figure S1. Study design. This study included 485 of 584 patients from 3 Chinese institutions. DRS-BCR: deep radiomic signature of biochemical recurrence; RP: radical prostatectomy; PUTH: Peking university third hospital; BJFH: Beijing friendship hospital; PUPH: Peking university people's hospital; PCa: prostate cancer; MRI: magnetic resonance imaging; ADT: androgen deprivation therapy.

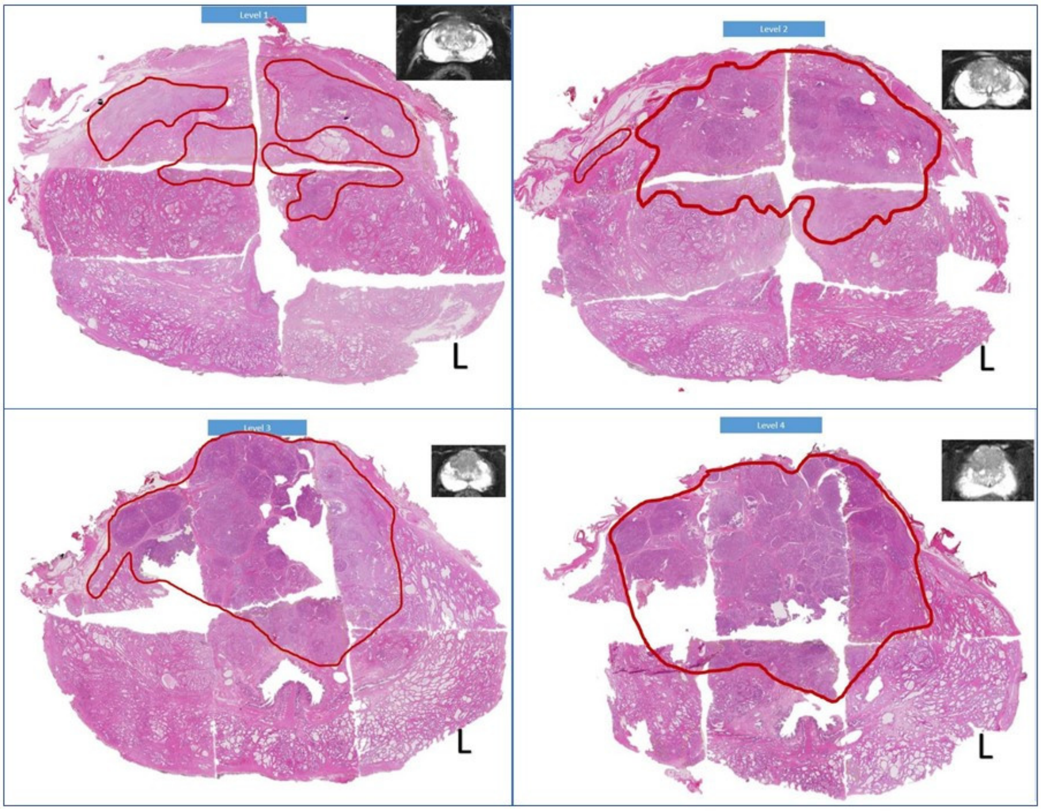

Figure S2. Detailed examples of annotations. Whole-mount slides of pathology sections were manually registered with T2 images. Generally, it is difficult to delineate prostate lesions solely based on MRI. By incorporating computational pathologic whole-mount slides to verify radiologic segmentations on each slice of MR imaging, it can make the current work more robust and reproducible.

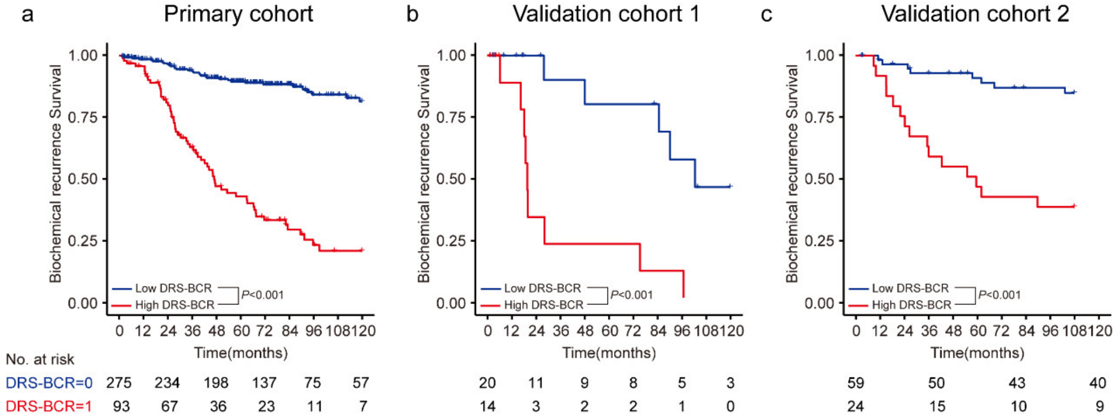

Figure S3. Performance of DRS-BCR for predicting 5-year BCR free survival. (a-c) The Kaplan-Meier (K-M) curves of DRS-BCR free survival for BCR free survival within five years in the primary cohort, validation cohort 1, and validation cohort 2, respectively. The p values were calculated by log-rank test between subgroup with high-risk and low-risk, and significant discrimination was revealed by p values less than 0.05. P-values were calculated using two-sided log-rank tests. DRS-BCR: deep radiomic signature of biochemical recurrence.

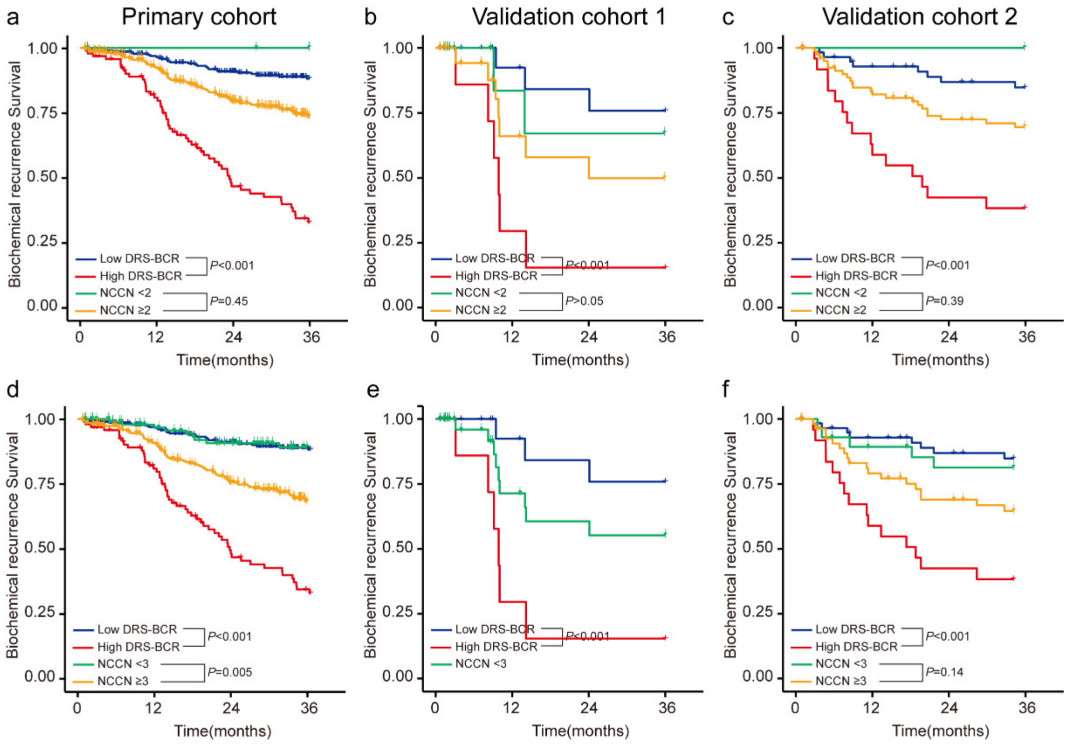

Copyright: © 2021 by the authors. Licensee MDPI, Basel, Switzerland. This article is an open access article distributed under the terms and conditions of the Creative Commons Attribution (CC BY) license (http://creativecommons.org/licenses/by/4.0/).

Figure S4. Comparisons between DRS-BCR and NCCN model by Kaplan-Meier (K-M) analysis. NCCN model (1, very low risk; 2, low risk; 3, intermediate risk; 4, high risk; 5, very high risk). (a-c) Comparison of a division of NCCN model (1 vs. others) in the primary cohort, validation cohort 1, and validation cohort 2. (d-f) Comparison of a division of NCCN model (1-2 vs. others). DRS: deep radiomic signature; NCCN: National Comprehensive Cancer Network.

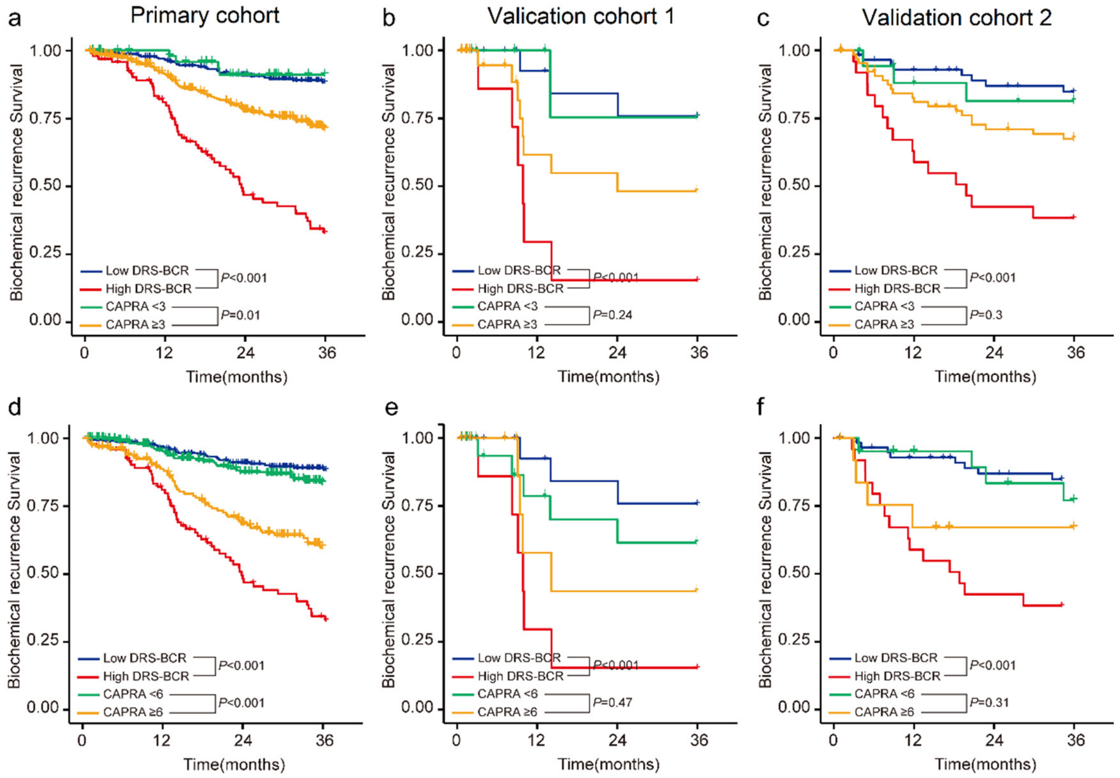

Figure S5. Comparisons between DRS-BCR and CAPRA score by Kaplan-Meier (K-M) analysis. CAPRA score (0-2, low risk; 3-5, medium risk; 6, high risk). (a-c) Comparison of a division of CAPRA score (0-2 vs. others) in the primary cohort, validation cohort 1, and validation cohort 2. (d-f) Comparison of a division of CAPRA score (0-5 vs. others). P-values were calculated using two-sided log-rank tests. DRS-BCR: deep radiomic signature of biochemical recurrence; CAPRA: Cancer of Prostate Risk Assessment.

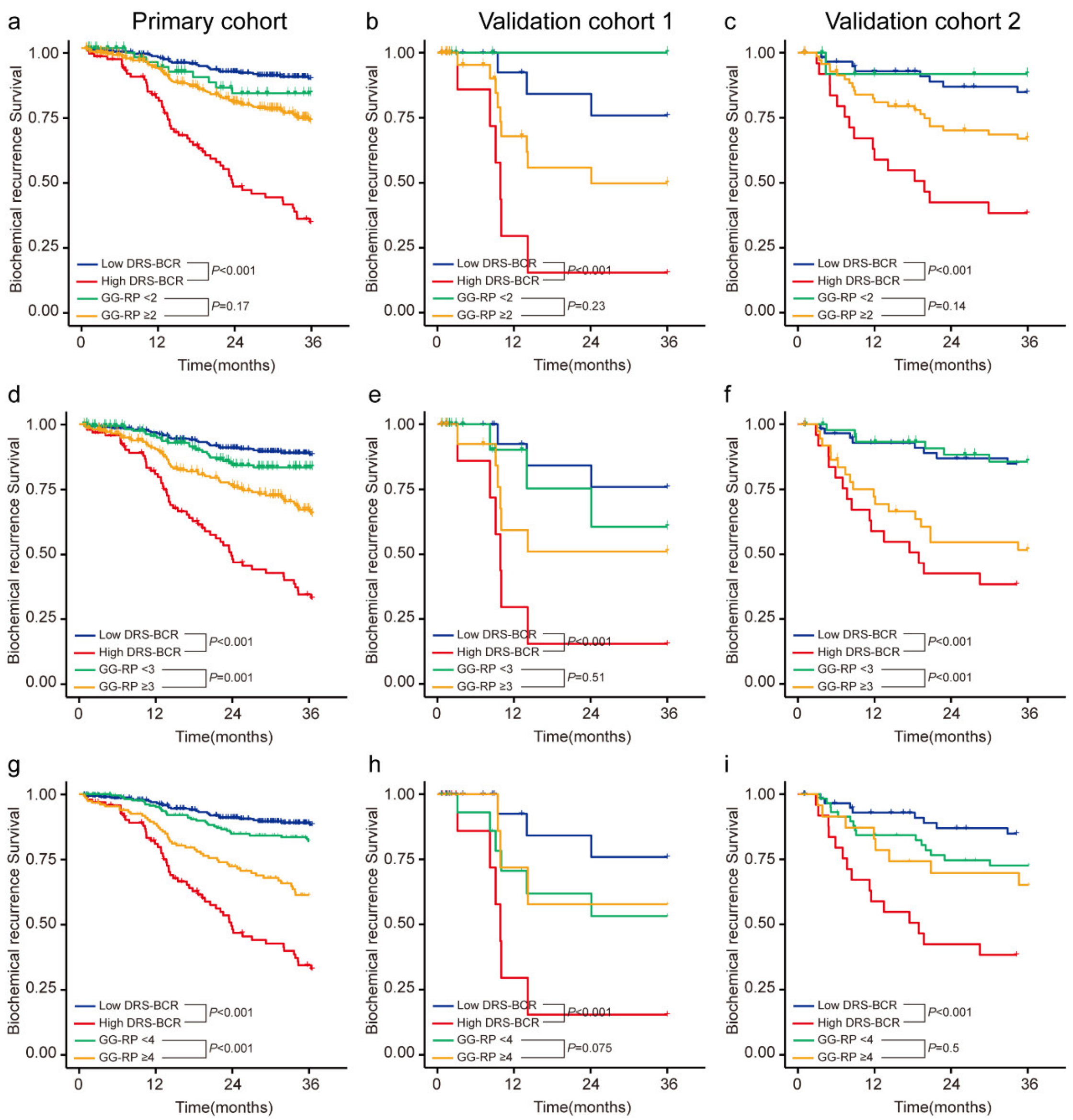

Figure S6. Comparisons between DRS-BCR and GG-RP by Kaplan-Meier (K-M) analysis. GG-RP (1, Gleason 3+3; 2, Gleason 3+4; 3, Gleason 4+3; 4, Gleason 4+4/3+5/5+3; 5, Gleason 4+5/5+4/5+5;). (a-c) Comparison of a division of GG-RP (1 vs. 2-5) in the primary cohort, validation cohort 1, and validation cohort 2. (d-f) Comparison of a division of GG-RP (1-2 vs. 3-5). (g-i) Comparison of a division of GG-RP (1-3 vs. 4-5). P-values were calculated using two-sided log-rank tests. DRS-BCR: deep radiomic signature of biochemical recurrence; GG-RP: Gleason grade group of radical prostatectomy.

Table S1. MRI Parameters.

	PUTH	BJFH	PUPH
Manufacturer	Siemens Healthcare，Erlangen, Germany	General Electric, Milwaukee, USA	General Electric, Milwaukee, USA
Model	3T Trio Tim	3T Discovery MR750	3T Discovery MR750
Coils	None	None	None
T2 weighted imaging			
Repetition Time/Echo Time	3290/95	4500/93	4000/127
Acquisition voxel size (mm3)	0.625*0.625*4.8	0.469*0.469*4.6	0.508*0.508*4.5
Acquisition time (min)	2.03	2.73	2.55
Diffusion weighted imaging			
Repetition Time/Echo Time	3600/80	4100/61	6050/56
Acquisition voxel size (mm3)	2.188*2.188*4.4	1.055*1.055*4.6	1.406*1.406*4.5
B-values (s/mm2)	0, 200, 800, 1000	0, 50, 800, 1500, 2000	0, 50, 400, 800, 1500
Acquisition time (min)	3.01	4.30	4.21

Note: PUTH, Peking University Third Hospital; BJFH: Beijing Friendship Hospital; PUPH, Peking University People Hospital.

Table S2. Univariate analysis of clinical factors.

	HRs [95% CI]	*P*	C-index		
			PC(N=369)	VC1(N=34)	VC2(N=83)
PSA	1.4<br>[1.2-1.6]	<0.001	0.636<br>[0.578-0.694]	0.59<br>[0.429-0.751]	0.616<br>[0.509-0.723]
GS-NB	1.786<br>[1.352-2.36]	<0.001	0.605<br>[0.553-0.657]	0.605<br>[0.452-0.758]	0.641<br>[0.538-0.744]
cT	2.228<br>[1.435-3.458]	<0.001	0.599<br>[0.553-0.645]	0.555<br>[0.419-0.691]	0.516<br>[0.421-0.611]
PPB	1.969<br>[1.29-3.006]	0.002	0.589<br>[0.54-0.638]	0.616<br>[0.474-0.758]	0.533<br>[0.442-0.624]
GS-RP	1.645<br>[1.217-2.224]	0.001	0.583<br>[0.53-0.636]	0.564<br>[0.419-0.709]	0.689<br>[0.601-0.777]
SM	1.803<br>[1.21-2.687]	0.003	0.587<br>[0.535-0.639]	0.631<br>[0.504-0.758]	0.515<br>[0.42-0.61]
EPE	1.952<br>[1.309-2.911]	0.001	0.588<br>[0.536-0.64]	0.512<br>[0.379-0.645]	0.504<br>[0.421-0.587]
SVI	2.748<br>[1.682-4.49]	<0.001	0.581<br>[0.535 0.627]	0.549<br>[0.434-0.664]	0.517-<br>[0.471 0.563]

Note: PSA (0, 2.1-6; 1, 6.1-10; 2, 10.1-20; 3, 20.1-30; 4,>30); GS-NB, Gleason score of needle biopsy (0, no Gleason patttern 4 or 5; 1, secondary Gleason pattern 4 or 5; 2, primary Gleason pattern 4 or 5); cT (0, T1/T2; 1, T3a); PPB, percentage of positive biopsy cores (0, <34%; 1, ≥34%); GS-RP, Gleason score of radical prostatectomy (0, 2-6; 1, 3+4; 2, 4+3; 3, 8-10); SM, surgical margin (0, negative; 1, positive); EPE, extracapsular extension (0, negative; 1, positive); SVI, seminal vesicle invasion (0, negative; 2, positive).

Table S3. Multivariate analysis of pre-operative clinical factors.

	**HRs [95% CI]**	***P***	**C-index**		
			**PC(N=369)**	**VC1(N=34)**	**VC2(N=83)**
Model			0.681 [0.624-0.738]	0.645 [0.466-0.824]	0.637 [0.525-0.749]
PSA	1.262 [1.073-1.484]	0.005			
GS-NB	1.463 [1.085-1.97]	0.013			
cT	1.727 [1.098-2.714]	0.018			
PPB	1.207 [0.7568-1.925]	0.43			

Note: PSA (0, 2.1-6; 1, 6.1-10; 2, 10.1-20; 3, 20.1-30; 4,>30); GS-NB, Gleason score of needle biopsy (0, no Gleason patttern 4 or 5; 1, secondary Gleason pattern 4 or 5; 2, primary Gleason pattern 4 or 5); cT (0, T1/T2; 1, T3a); PPB, percentage of positive biopsy cores (0, <34%; 1, ≥34%).

Table S4. Multivariate analysis of post-operative clinical factors.

	HRs [95% CI]	*P*	C-index		
			**PC(N=369)**	**VC1(N=34)**	**VC2(N=83)**
Model			0.652 [0.592-0.712	0.558 [0.389-0.727]	0.57 [0.472-0.668]
GS-RP	1.393 [1.013-1.917]	0.041			
SM	1.465 [0.967-2.22]	0.071			
EPE	1.208 [0.739-1.975]	0.451			
SVI	1.904 [1.07-3.388]	0.028			

Note: GS-RP, Gleason score of radical prostatectomy (0, 2-6; 1, 3+4; 2, 4+3; 3, 8-10); SM, surgical margin (0, negative; 1, positive); EPE, extracapsular extension (0, negative; 1, positive); SVI, seminal vesicle invasion (0, negative; 2, positive).

Table S5. Multivariate analysis of combination of perioperative clinical factors (CS-combine).

	HRs [95% CI]	*P*	C-index		
			PC(N=369)	VC1(N=34)	VC2(N=83)
model			0.693 [0.634-0.752]	0.651 [0.471-0.831]	0.641 [0.529-0.753]
PSA	1.228 [1.039-1.451]	0.016			
GS-NB	0.967 [0.667-1.404]	0.862			
cT	1.642 [1.031-2.615]	0.037			
PPB	1.086 [0.666-1.770]	0.742			
GS-RP	1.418 [1.002-2.008]	0.049			
SM	1.264 [0.824-1.937]	0.283			
EPE	1.028 [0.619-1.707]	0.916			
SVI	1.627 [0.901-2.938]	0.106			

Note: PSA (0, 2.1-6; 1, 6.1-10; 2, 10.1-20; 3, 20.1-30; 4,>30); GS-NB, Gleason score of needle biopsy (0, no Gleason pattern 4 or 5; 1, secondary Gleason pattern 4 or 5; 2, primary Gleason pattern 4 or 5); cT (0, T1/T2; 1, T3a); PPB, percentage of positive biopsy cores (0, <34%; 1, ≥34%); GS-RP, Gleason score of radical prostatectomy (0, 2-6; 1, 3+4; 2, 4+3; 3, 8-10); SM, surgical margin (0, negative; 1, positive); EPE, extracapsular extension (0, negative; 1, positive); SVI, seminal vesicle invasion (0, negative; 1, positive).

Table 6. Statistic of selected features from radiomic features.

N (%)	First-order	Shape	Texture
Sum	22 (14.2%)	8 (5.16%)	125(80.6%)
Origin	2	8	21
LoG	10		48
sigma=0.1	5		20
sigma=0.3	3		14
sigma=0.5	2		14
Wavelet	10		56
HH	2		10
HL	3		22
LH	4		8
LL	1		16

The features pool consisted of (i) 18 first-order features, (ii) 14 shape features, (iii) 68 texture features (GLDM, GLCM, GLRLM, GLSZM), (iv) 344 wavelet features (HH, HL, LH, and LL), and (v) 258 Laplacian of Gaussian (LoG) filter features (sigma value were 1.0, 3.0 and 5.0). The wavelet features and LoG filter features, including two categories of features (first-order and textures), were extracted from images with corresponding image processing methods to expand original features to high dimension features. From concordance, there were 636 (90.7%) features showed potential correlation with BCR (C-index>0.5). From significance evaluation, a total of 160 (22.8) features in the pool demonstrated significant differences between BCR and non-BCR status (P<0.05).

Table S7. Evaluation of DRC-BCR for BCR survival.

	HRs [95% CI]	P	C-index		
			PC(N=369)	VC1(N=34)	VC2(N=83)
model			0.807 [0.76-0.854]	0.794 [0.685-0.903]	0.8 [0.723-0.877]
DRS-BCR	1.654 [1.486-1.842]	<0.001			
PSA	1.13 [0.959-1.332]	0.146			
GS-RP	1.286 [0.947-1.746]	0.107			
EPE	1.472 [0.957-2.264]	0.078			

Note: PSA (0, 2.1-6; 1, 6.1-10; 2, 10.1-20; 3, 20.1-30; 4,>30); DRC-BCR: deep radiomic combing signature of biochemical recurrence; GS-RP, Gleason score of radical prostatectomy (0, 2-6; 1, 3+4; 2, 4+3; 3, 8-10); EPE, extracapsular extension (0, negative; 1, positive).

Table 8. Comparison between DRC-BCR and DRS-BCR.

		PC (N=369)	VC1 (N=34)	VC2 (N=83)
U-statistics-based C estimator	Difference	0.005	-0.017	0.005
	P	0.539	0.719	0.614
Wilcoxon signed rank test	*P*	0.691	0.782	0.941
IDI test	IDI	0.014 [-0.109-0.182]	0.014 [-0.113-0.192]	0.016 [-0.040-0.075]
	P	0.945	0.886	0.488
continuious-NRI test	continuious-NRI	0.176 [-0.527-0.606]	0.176 [-0.423-0.729]	0.037 [-0.407 0.538]
	P	0.935	0.826	0.706

DRC-BCR: deep radiomic combing signature of biochemical recurrence; DRS-BCR: deep radiomic signature of biochemical recurrence.

Table 9. Comparison between DRS-BCR and GG-RP.

		PC(N=369)	VC1(N=34)	VC2(N=83)
U-statistics-based C estimator	Difference	0.219	0.247	0.106
	P	<0.001	0.002	0.036
IDI test	IDI	0.316 [0.213-0.409]	0.395 [0.111-0.651]	0.177 [0-0.356]
	P	<0.001	0.021	0.049
continuious-NRI test	continuious-NRI	0.412 [0.251-0.571]	0.460 [0.000-0.834]	0.325 [-0.003-0.576]
	P	<0.001	0.049	0.05

Note: difference = C-index of DRS-BCR - C-index of GG-RP. DRS-BCR: deep radiomic signature of biochemical recurrence; GG-RP: Gleason grade group of radical prostatectomy.

Table 10. Comparison between DRS-BCR and CAPRA-S.

		PC(N=369)	VC1(N=34)	VC2(N=83)
U-statistics-based C estimator	Difference	0.125	0.157	0.140
	P	<0.001	0.121	0.005
IDI test	IDI	0.244 [0.133-0.343]	0.310 [-0.046-0.616]	0.236 [0.081-0.373]
	P	<0.001	0.091	<0.001
continuious-NRI test	continuious-NRI	0.443 [0.265-0.571]	0.560 [0.000-0.858]	0.544 [0.165-0.669]
	P	<0.001	0.048	<0.001

Note: difference = C-index of DRS-BCR - C-index of CAPRA-S. DRS-BCR: deep radiomic signature of biochemical recurrence.

Table 11. Comparison between DRS-BCR and NCCN.

		PC(N=369)	VC1(N=34)	VC2(N=83)
U-statistics-based C estimator	Difference	0.216	0.276	0.211
	P	<0.001	<0.001	<0.001
IDI test	IDI	0.306 [0.209-0.401]	0.393 [0.039-0.637	0.267 [0.130-0.372]
	P	<0.001	0.024	<0.001
continuious-NRI test	continuious-NRI	0.437 [0.294-0.567]	0.560 [-0.024-0.893]	0.413 [0.198-0.650]
	P	<0.001	0.052	<0.001

Note: difference = C-index of DRS-BCR - C-index of NCCN. DRS-BCR: deep radiomic signature of biochemical recurrence.

Table 12. Comparison between DRS-BCR and CAPRA.

		**PC(N=369)**	**VC1(N=34)**	**VC2(N=83)**
U-statistics-based C estimator	Difference	0.125	0.259	0.180
	P	<0.001	0.014	<0.001
IDI test	IDI	0.238 [0.124-0.342]	0.397 [0.022-0.668]	0.282 [0.123-0.423]
	P	<0.001	0.029	<0.001
continuious-NRI test	continuious-NRI	0.413 [0.213-0.575]	0.560 [0.000-0.870]	0.470 [0.225-0.656]
	P	<0.001	0.047	<0.001

Note: difference = C-index of DRS-BCR - C-index of CAPRA. DRS-BCR: deep radiomic signature of biochemical recurrence.

Table S13. Fourfold comparison between DRS-BCR/CAPRA-S with true BCR events.

		True BCR+	True BCR-	Sensitivity	Specificity	PPV	NPV
DRS-BCR model	High Risk	75	53	0.664	0.858	0.586	0.894
	Low Risk	38	319				
CAPRA-S model	High Risk	95	260	0.841	0.301	0.268	0.862
	Low Risk	18	112				

PPV: positive predictive value; NPV: negative predictive value. DRS-BCR: deep radiomic signature of biochemical recurrence.

I. Incorporating radiomic signature with clinical variables

Multivariate analyses were carried out among perioperative clinical factors including PSA-level, grade group of needle biopsy (GG-NB), clinical T stage (cT), percentage of positive biopsy cores (PPB), grade group of RP (GG-RP), surgical margin (PSM), extracapsular extensions (EPE) and seminal vesicle extensions (SVI). The DRS-BCR was further evaluated in combination with afore-mentioned clinical factors to develop a deep radiomic combing model for prediction of BCR (DRC-BCR). Perioperative parameters were normalized by CAPRA [1] and CAPRA-S [2] criteria. The stepwise strategy [3] was employed to select meaningful predictors by the Akaike information criterion for modeling. The DRC-BCR was then validated in VC1 and VC2.

II. Comparison between conventional clinical models and DRS-BCR

The GG-RP model, CAPRA/CAPRA-S score and NCCN model were applied to stratify patients into high-risk (including intermediate-risk group) and low-risk groups for 3-year BCR estimation, respectively. The Kaplan-Meier (K-M) analysis was used to evaluate the performance of each model. The p-value of the log-rank test was used to evaluate the discriminative significance ($P<0.05$). We compared DRS-BCR and other models by adopting the U-statistics-based C estimator and developed a nonparametric analytical approach to estimate the variance of the C estimator and the covariance of two C estimators [4]. The integrated discrimination improvement (IDI) and continuous net reclassification improvement (continuous-NRI) was used to evaluate improvement of DRS-BCR's performance [5].

III. Performance of incorporating DRS-BCR with clinical variables

By stepwise strategy of multivariable logistic regression model using both DRS-BCR and clinical variables, DRS-BCR, PSA, EPE, and GS-RP were included to construct deep radiomic combing signature for BCR-survival (DRC-BCR). The C-index [95% CI] of DRC-BCR were 0.807 [0.76-0.854] in PC, 0.794 [0.685-0.903] in VC1, and 0.8 [0.723-0.877] in VC2 (**Table S6**). Within the model, compared with clinical variables, DRS-BCR was the only parameter achieving independent significance. Additionally, C-index distributions and reclassification performances of DRS-BCR and DRC-BCR did not show any significant difference (**Table S7, S8**).

IV. More comparison between DRS-BCR and conventional clinical predictive models

Comparisons between DRS-BCR with CAPRA and GG-RP by K-M analysis were shown in Figure S5 and Figure S6.

Additionally, we carried out a detailed comparison between DRS-BCR and CAPRA-S system by using classic fourfold table (Table S13). Comparing to CAPRA-S model, DRS-BCR signature significantly increased specificity (0.858 vs. 0.301), positive predictive value (0.586 vs. 0.268) and maintained negative predictive value at a high level of 0.894. As a consequence of compensation, sensitivity of the DRS-BCR model slightly reduced to 0.664.

References

1. Cooperberg, M.R., et al., *The University of California, San Francisco Cancer of the Prostate Risk Assessment score: a straightforward and reliable preoperative predictor of disease recurrence after radical prostatectomy.* J Urol, 2005. **173**(6): p. 1938-42.
2. Cooperberg, M.R., J.F. Hilton, and P.R. Carroll, *The CAPRA-S score: A straightforward tool for improved prediction of outcomes after radical prostatectomy.* Cancer, 2011. **117**(22): p. 5039-46.
3. Hesterberg and Tim, *Statistical Models in S.* Technometrics, 2012. **35**(2): p. 227-228.
4. Kang, L., et al., *Comparing two correlated C indices with right-censored survival outcome: a one-shot nonparametric approach.* Stats in Medicine, 2015. **34**(4).
5. Kerr, K.F., et al., *Evaluating the Incremental Value of New Biomarkers With Integrated Discrimination Improvement.* American Journal of Epidemiology, 2011. **174**(3): p. 364-374.
